# Wearable and Thermal Drift-Compensated Monitoring System Based on Fiber Bragg Grating Sensors for a 3D-Printed Foot Prosthesis

**DOI:** 10.3390/s25030885

**Published:** 2025-01-31

**Authors:** Sara Del Chicca, Gennaro Rollo, Andrea Sorrentino, Emanuele Gruppioni, Marco Tarabini, Paola Saccomandi

**Affiliations:** 1 Dipartimento di Meccanica, Politecnico di Milano, Via Privata Giuseppe La Masa 1, 20156 Milan, Italy; sara.delchicca@polimi.it (S.D.C.);; 2 Institute of Polymers, Composites and Biomaterials, National Research Council, Via Previati 1/E, 23900 Lecco, Italy; gennaro.rollo@ipcb.cnr.it (G.R.); andrea.sorrentino@cnr.it (A.S.); 3 Centro Protesi, INAIL, Via Rabuina, 14, 40054 Vigorso di Budrio, Italy; e.gruppioni@inail.it

**Keywords:** compensation algorithm, FBG sensor, Fiber Bragg Grating, foot prosthesis, light source instability, monitoring, optoelectronic unit, signal drift

## Abstract

Monitoring foot prostheses is essential, as their performance impacts users’ daily lives. Fiber Bragg Grating (FBG) sensors represent a gold standard in monitoring applications, but traditional optoelectronic units are too cumbersome for wearable applications. This research addresses this issue by using a lightweight and compact optoelectronic unit and developing a compensation algorithm to overcome the signal drift phenomena caused by the light source instability. The proposed method uses an FBG as a reference to provide the algorithm with information on the signals drift. The developed algorithm is based on the assumptions of linearity among drift in different detection channels and the absence of drift at the initial time instant. The compensation variable was experimentally identified and validated. Experimental validation through temperature tests showed the algorithm reduces the drift error by 60%. Finally, mechanical tests were conducted on a foot prosthesis equipped with two FBGs: one used as a reference and the other for strain sensing. An electrical strain gauge was used to validate the FBG-based sensing system. The results of the mechanical tests indicate the possiblity to monitor a foot prosthesis using FBGs. The FBG and strain gauge measurements comparison aligns with previous studies where high-performance optoelectronic units were used.

## 1. Introduction

Amputation is a major cause of disability, drastically reduces physical functions, and represents a significant global health economic burden in recent decades. In 2017, the number of amputees worldwide reached 57.7 million [1]. Among the possible amputations, unilateral lower limb amputation causes the greatest burden of disability [2]. These data motivate the need for continuous innovation in the prosthesis field to improve patients’ daily lives, and the necessary steps for this improvement are innovative and cost-effective approaches to prosthesis manufacturing and system performance monitoring. Nowadays, prosthesis performances are not evaluated during their lifetime: commercial prosthesis monitoring is mainly qualitative [3], and the lifetime is scheduled. In the research field studies have been conducted to monitor lower-limb prostheses, however, they are restricted to a laboratory or clinical environment [3]. Implementing monitoring technologies in prostheses could increase the knowledge of practitioners and improve care methods [4]. Sensors selection in this contest is a critical phase [5]. Such monitoring systems need to be lightweight, provide easy integration, and have high resolution. Fiber Bragg Grating (FBG) sensors possess all the requirements, and are widely used in monitoring fields [6]. Moreover, FBGs are characterized by flexibility, embeddability, small size/weight, immunity to electrical or magnetic interference [7,8], and bio-compatibility. Due to their characteristics, the use of FBG sensors in the biomedical field has increased in recent years [9]. Applications of FBGs in prosthetics concern the sensorization of the socket to improve the interface between the residual limb and the prosthesis [10,11].

An interesting example for the presented application is the work of Glav$\tilde{a}$o [12], in which a composite foot prosthesis was sensed with 9 FBGs for force and temperature measurement at different locations and then tested on a treadmill. The authors showed that it is possible to multiplex several FBGs along a single optical fiber for a distributed measurement throughout the prosthesis. In particular, the best results came from the FBGs located in the region where the prosthesis stores and releases energy during walking. In [13] it is discussed the possibility of integrating FBG sensors in the insole of a foot prosthesis to provide gait control, terrain identification, and mimic the human sense of touch. In [14] four FBG sensors were embedded in a carbon fiber-reinforced polymer foot and the acquired signals were used to control the smart foot implementing a Fuzzy Logic control strategy. Results demonstrated an improvement in the control proprioception. In the present work, a new sensorized foot prosthesis made of composite material is being developed: its fabrication is based on the innovative additive manufacture process of continuous filament deposition of the reinforcing fiber within the composite. The prosthesis is equipped with FBG sensors to monitor its performance over time and utilization. Therefore, one of the main challenges is to implement a wearable FBG-based system on a foot prosthesis.

The main limitation of FBG-based sensing systems in this field of application is the optoelectronic unit required to manage the sensors. Standard interrogation units can not be portable due to their dimensions [15]. Also, when small, the power supply and processing unit requirements restrict their portability. There is some research ongoing regarding the development of completely portable FBG interrogation devices for gait detection [16] or plethysmography application [17]. In this context, the presented work aims to implement a wearable monitoring system based on FBG sensors in a 3D-printed foot prosthesis.

The optoelectronic unit examined in the study is the Miniature MOFIS by Redondo Optics Inc. described in [18]. It is wireless, lightweight, and compact, marking an advancement in everyday applicability. However, these favorable qualities come with the drawback of light source instability, leading to wavelength drift in the acquired signals. To ensure measurement stability and accuracy, and to address issues related to drift, a compensation algorithm was developed and tested. Signal drift is a well-documented challenge in the field of optoelectronic units for FBG sensors [19]. Researchers have investigated various strategies to address this issue, covering both hardware and software approaches. From a software perspective, many solutions leverage Artificial Intelligence (AI) algorithms. For instance, in [20], an Adaptive Weight Least Squares Support Vector Regression was employed to deal with drift and hysteresis effects. Similarly, in [21], a hybrid approach combining a Convolutional Neural Network with a Long Short-Term Memory Neural Network was developed to predict and correct signal drift. While AI-based methods are powerful and promising, they are often computationally intensive, making them unsuitable for certain applications, including the one presented here. An alternative solution proposed in [22] utilizes two FBG sensors to address drift; however, the non-linear relationship between voltage and intensity limits its applicability in our specific context.

The following sections are organized as follows. Firstly, a brief overview of the operating principles of FBGs and the interrogation unit under consideration is presented. Subsequently, the compensation algorithm is described. Tests were conducted to evaluate the performance of the designed measurement system. Specifically, three distinct tests were carried out. The initial test was conducted under static conditions for algorithm development. The second test involved exposing the system to static temperatures within a thermal chamber to validate the stability of the signal after the compensation procedure. Finally, a mechanical test was performed on the instrumented prosthesis.

## 2. Materials and Methods

### 2.1. Working Principle of Fiber Brag Grating Sensors

The sensing principle of an FBG is based on a periodic perturbation of the refractive index along the fiber length caused by a photo-incision of the optical fiber core. Grating is characterized by its spatial period Λ, and the effective refractive index, $n_{eff}$ [23]. The FBG acts like a stop band filter [24]: when a broadband light source interrogates the FBG, a narrow band of the incident optical field is reflected by successive coherent scattering from the index variations [25]. The reflected narrow band is centered at the Bragg wavelength [26,27]:

(1) $$\lambda_{B}=2n_{eff}\Delta$$

FBG can be used to measure temperature and strain. When the optical fiber is stretched the related deformation changes grating period Λ and thus the Bragg wavelength [28]. Instead, when the optical fiber is heated the main contribution to the change in wavelength is the alteration in silica refraction index, induced by the thermo-optical effect. The contribution of thermal expansion is marginal, given silica’s low thermal expansion coefficient [28]. The shift of the Bragg wavelength (Δλ) form the unstretched condition ($\lambda_{B0}$) follows the mechanical strain ϵ and the temperature variation ΔT as reported in (2):

(2) $$\frac{\Delta \lambda }{\lambda_{B0}}=k\cdot \epsilon +\alpha_{T}\cdot \Delta T$$

where *k* is the gauge factor that considers the photo-elastic coefficient and $\alpha_{T}\;\left[\mathrm{nm}\cdot K^{-1}\right]$ is the global thermal coefficient that considers the thermal expansion coefficient of the fiber and the fiber bonding material and the thermal-optic coefficient. The analytical expressions for the mechanical strain inducing a wavelength change can be obtained directly from (2):

(3) $$\epsilon =\frac{1}{k}\frac{\Delta \lambda }{\lambda_{B0}}-\frac{\alpha_{T}}{k}\Delta T$$

### 2.2. Optoeletronic Unit: Redondo Optics Miniature MOFIS

Redondo Optics Inc. miniature MOFIS^*TM*^ is a fully integrated FBG interrogation device. It can monitor sensor transducers distributed along a single optical fiber with a built-in intensity reference monitor. MOFIS device is characterized by:

- a broadband light source in the C-band 1550 nm window;
- four individual high-sensitive detection channels;
- a switching technology to acquire multiple fibers in sequential mode.

**Figure 1:**
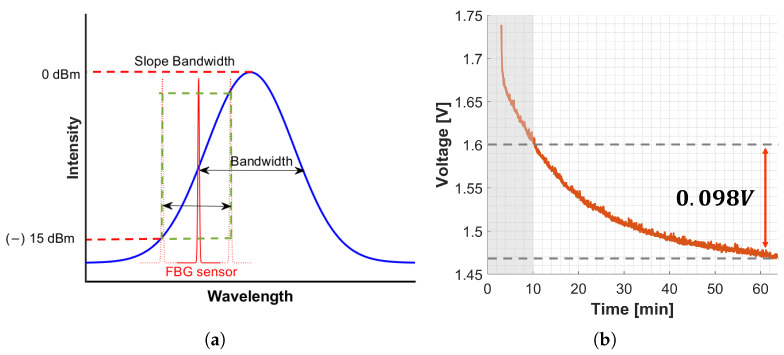
(**a**) Working principle of the integrated Gaussian WDM demodulator adapted from [31]. (**b**) Voltage signal of undisturbed FBG affected by drift.

**Light Source.** MOFIS^*TM*^ employs a Superluminescent Light Emitting Diode (SLED) for light emission. SLEDs are semiconductor devices emitting broadband light through electrical current injection, representing a hybrid technology between LEDs and laser diodes.
**Monitoring Mode.** MOFIS^*TM*^ monitoring mode is based on Wavelength Division Multiplexing (WDM), a technology that multiplexes several optical carrier signals onto a single optical fiber using different wavelengths of laser light [29,30]. WDM enables the sharing of an optical fiber among many different signals simultaneously. The WDM operating principle for an array of FBGs works as follows: the SLED light is sent along the fiber and each FBG reflects a particular wavelength $\lambda_{B}$. Then, the reflected spectrum is analyzed via WDM Gaussian demodulators. The demodulators output a voltage value that depends on the wavelength returned. Figure 1a shows the working principle of the WDM demodulator integrated into the optoelectronic unit under study. The provided software implements a Gaussian model to fit the intensity to wavelength response of the WDM demodulator. The mathematical equation describing the model is provided in (4):
  (4) $$V=V_{max}e^{-\frac{{\left(\lambda -\lambda_{c}\right)}^{2}}{2c^{2}}}+V_{offset}$$
  where *V* is the measured voltage and λ is the Bragg wavelength corresponding to the measured voltage. The model is defined by the optical filter central wavelength $\lambda_{c}$ and the optical filter standard deviation *c*, related to the full-width-half-maximum of the filter.

Table 1 resumes the engineering specification of the device under study.

### 2.3. Compensation Algorithm

Drift affects MOFIS^*TM*^ signals, as shown in Figure 1b. The signal shows a constant descending trend, even though the FBG connected to the device is not subject to temperature variation or deformation. The light source drift caused a signal drop of 98 mV, corresponding to 200μϵ equal to 10% of the maximum readable strain. This behavior is addressed by overheating the electronic components that begins when the MOFIS*^TM^* is turned on and leads to the instability of the light source. A model is developed to compensate for this drift to ensure measurement accuracy.

MOFIS*^TM^* is equipped with four sensing channels, so it is possible to compensate for the drift by using one channel as a reference and the other three as sensors. From now on, the subscript R will refer to the channel compatible with the FBG adopted as a reference, and the subscript S will refer to the channel compatible with the FBG adopted as a sensor. In this study the reference channel, i.e., R, corresponds to the detection channel number one of the MOFIS^*TM*^ optoelectronic unit; instead, the sensing, i.e., S, channel corresponds to the detection channel number four of the MOFIS^*TM*^ optoelectronic unit.

Considering the presence of drift, it is possible to split the measured voltage ($V_{X_{meas}}$) of each detection channel into three contributions.

1. ${\tilde{V}}_{X}\left(\epsilon ,\Delta T\right)$ is the informative contribution of the measured voltage. $\tilde{V}$ is the measured voltage share due to the wavelength shift caused by strain or temperature variation.
2. $V_{X_{offset}}$ is the voltage offset due to electronics, it is constant and measured before plugging in the fiber.
3. $V_{X_{drift}}$ is the measured voltage share caused by light source instability.

where subscript _*X*_ must be replaced by _*R*_ when referring to the reference channel or by _*S*_ when referring to the sensing channel. Equation (5) resume these concepts:

(5a) $$\begin{array}{c} V_{S_{meas}}={\tilde{V}}_{S}\left(\epsilon ,\Delta T\right)+V_{S_{offset}}+V_{S_{drift}} \end{array}$$

(5b) $$\begin{array}{c} V_{R_{meas}}={\tilde{V}}_{R}\left(\epsilon ,\Delta T\right)+V_{R_{offset}}+V_{R_{drift}} \end{array}$$

Two hypotheses were considered to develop the model for drift compensation:

- **Hp1:** the drift voltage share in the detection channel adopted as the sensor is directly proportional to that of the detection channel adopted as a reference, as shown in (6a). **Hp1** is a fundamental assumption for the proposed method. Its validity was experimentally verified by calculating the Pearson correlation coefficient on the signals acquired from the reference and sensing FBGs under resting conditions, Section 2.5.1. The Pearson coefficient was calculated with the standard formula reported in (6b):
  (6a) $$\begin{array}{c} V_{S_{drift}}=k*V_{R_{drift}} \end{array}$$
  (6b) $$\begin{array}{c} \rho_{Hp1}=\frac{Cov(V_{R_{drift}},V_{S_{drift}})}{\sigma_{V_{R_{drift}}},\sigma_{V_{S_{drift}}}} \end{array}$$
  where $Cov(V_{R_{drift}},V_{S_{drift}})$ is the covariance between $V_{R_{drift}}$ and $V_{S_{drift}}$; whereas $\sigma_{V_{R_{drift}}}$ and $\sigma_{V_{S_{drift}}}$ are the standard deviation of $V_{R_{drift}}$ and $V_{S_{drift}}$, respectively.
- **Hp2:** there is no drift at the initial instant, so the voltage share due to drift is null at *t* = 0 for both detection channels:
  (7a) $$\begin{array}{c} V_{R_{drift}}\left(t=0\right)=0V \end{array}$$
  (7b) $$\begin{array}{c} V_{S_{drift}}\left(t=0\right)=0V \end{array}$$

Thus, considering (7a) and (5b), it is possible to state that at the initial instant:

(8) $$\begin{array}{c} V_{R_{meas}}\left(t=0\right)={\tilde{V}}_{R}\left(\epsilon \right)+V_{R_{offset}}=V_{R_{uns}} \end{array}$$

where $V_{R_{uns}}$ is the voltage measured at t=0 in *unstretched* conditions when no strain or temperature variation is applied on the reference FBG. Therefore, if no drift occurs, the measured voltage during the test would be constant and equal to the initial ($V_{R_{uns}}$). That consideration allows for the calculation of the drift contribution in the reference detection channel.

Now imposing ***Hp1***, it is possible to isolate the informative voltage share (${\tilde{V}}_{S}\left(\epsilon ,\Delta T\right)$) starting from the measured one. After some calculations, it ends up as follows:

(9) $$\begin{array}{cc} {\tilde{V}}_{S}\left(\epsilon ,\Delta T\right) & =V_{S_{meas}}-V_{S_{offset}}-V_{S_{drift}}=\\ \; & =V_{S_{meas}}-V_{S_{offset}}-k\cdot V_{R_{drift}}=\\ \; & =V_{S_{meas}}-V_{S_{offset}}-k\cdot \left(V_{R_{meas}}-V_{R_{uns}}\right) \end{array}$$

The compensation variable k was identified through linear regression by relating the voltage measured on the sensing channel ($V_{S_{meas}}$) to the voltage measured on the reference channel ($V_{R_{meas}}$):

(10) $$\begin{array}{cc} V_{S_{meas}} & =\;{\tilde{V}}_{S}\left(\epsilon ,\Delta T\right)+V_{S_{offset}}+V_{S_{drift}}=\\ \; & =\;{\tilde{V}}_{S}\left(\epsilon ,\Delta T\right)+V_{S_{offset}}+\\ \; & \;+\;k\cdot (V_{R_{meas}}-{\tilde{V}}_{R}\left(\epsilon ,\Delta T\right)-V_{R_{offset}})=\\ \; & =\;k\cdot V_{R_{meas}}+\;{\tilde{V}}_{S}\left(\epsilon ,\Delta T\right)+\;V_{S_{offset}}-\\ \; & \;-\;k\cdot ({\tilde{V}}_{R}\left(\epsilon ,\Delta T\right)+\;V_{R_{offset}}) \end{array}$$

Summarizing:

(11) $$V_{S_{meas}}=k\cdot V_{R_{meas}}+b$$

where:

(12) $$\begin{array}{cc} b & ={\tilde{V}}_{S}\left(\epsilon ,\Delta T\right)+V_{S_{offset}}-k\cdot \left({\tilde{V}}_{R}\left(\epsilon ,\Delta T\right)+V_{R_{offset}}\right) \end{array}$$

The least-square-based identification method was applied to perform the identification of parameter ***k***. The signals for performing linear regression were acquired maintaining the two FBGs in rest condition for the entire test duration. The results of the identification procedure are shown in Section 3.

### 2.4. Sensors Positioning Identification

Two aspects were evaluated to identify the possible location for the FBG sensors: the characteristics and limitations of the sensing system and the measurement purpose. MOFIS*^TM^* allows sensing a percentage strain of 0.2% in each detection channel. It is possible to increase this range by using FBGs with a Bragg wavelength on the boundary of the Gaussian filter. Regarding FBGs, preliminary tests have shown that the maximum measurable strain in a tensile test without sensor breakage or sensor slippage phenomena is $\epsilon_{max}\%=0.5\%$. From the literature, the FBGs support compression up to $\Delta \lambda_{B}=-0.18$ nm [32].

**Figure 2:**
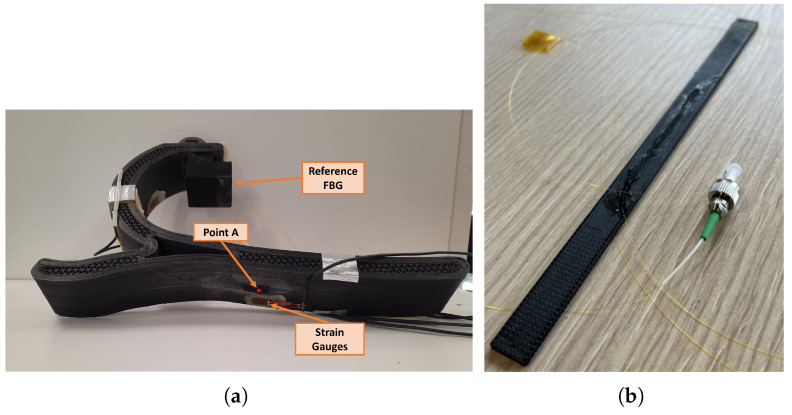
(**a**) Illustration of the sensorized prosthesis. (**b**) Carbon-fibre reinforced composite specimens with glued FBG sensor.

In terms of application, the scope of monitoring is the early detection of prosthesis failure and the evaluation of prosthesis performance. Therefore, it is necessary to identify the most informative locations on the prosthesis. Based on previous studies [33], the plantar area appears to be the one of interest for deformation monitoring, therefore a point is selected in this region (point A in Figure 2a) to explore the performance of the measurement system during prosthesis characterization. The reference FBG should be placed on a point not subject to deformation during the step phases. Analysis of the FEM study showed that no point meets this requirement on the prosthesis. To overcome this issue, it was decided to attach a 3D-printed holder to the prosthesis for positioning the reference FBG, as Figure 2a shows. The 3D printed support is intended to isolate the reference FBG from deformation.

### 2.5. Tests

#### 2.5.1. Parameter Identification and Algorithm Validation

Tests for the identification of the compensation variable k and validation of the proposed algorithm were performed using two single FBG optical fibers, each glued to a sample. The parameters of the used optical fibers are reported in Table 2.

Both specimens are characterized by a length *l* of 200 mm. The used matrix is *Onyx^TM^*, while the reinforcing element is carbon (CF) continuous fiber filament. Figure 2b shows a sensorized sample involved in these tests.

The test procedure was as follows:

- $V_{offset}$ was measured 10 min later the MOFIS was switched on.
- Optical fibers were plugged in after the $V_{offset}$ measurement.
- The test started 15 min after the fiber was plugged in.
- No temperature variation or strain were applied to the FBGs.
- Tests were performed on different days to be consistent.

#### 2.5.2. Temperature Test

Temperature tests were performed using a thermal box. This device allows setting and maintaining the temperature to a reference one. The settling time is about 10 min. This thermal box uses a PT100 sensor to measure the box temperature.

The sensing FBG was placed inside the box near the PT100 sensor. Instead, the reference FBG was placed outside the box to guarantee no temperature variation and no strain for the entire test duration. The procedure followed for the thermal test was:

- $V_{offset}$ was measured 10 min after the MOFIS was turned on.
- Optical fibers were plugged in after $V_{offset}$ measurement.
- The test started 15 min after the fiber was plugged in, and once the target temperature in the thermal chamber has been reached.

The thermal box temperature was set to 27 °C to perform the static test. The goal was to check the correct functioning of the drift compensation technique.

#### 2.5.3. Mechanical Tests

The response of the prosthesis during a mechanical test was acquired with an FBG glued to the identified point A (Figure 2a). To validate the response of the FBG, a strain gauge in a half-bridge configuration was placed near the FBG to verify the results. The strain gauges were calibrated using a known resistance and acquired with the SCOUT55 amplifier (Hottinger Brüel & Kjaer GmbH, Darmstadt, Germany).

The tests were carried out following the ISO 16955 [34] which describes the method to evaluate performance indicators of prosthetic foot devices. For testing the geometrical configuration of the foot, the platform, and the loading profile can be derived from ISO 22675 [35]. Specifically, the heel, mid-foot, and toe characteristics quantification procedure was applied in the simulation for identifying FBG positioning. Furthermore, the procedure was applied during the tests to validate the implemented FBG acquisition system.

Since the procedure is defined for a prosthetic foot that is 1.5 times bigger than the tested one, the loads were rescaled accordingly. The 16955 ISO states that the peak profile in the loading shall be 200% of the imposed body weight for mid-stance (0°) and 120% of the imposed body weight for inclinations other than 0°. Table 3 reports the three tested inclinations and the related loads. Positive inclinations refer to toe-supporting conditions. For each condition, the load was first applied in a quasi-static manner, with a machine crossbar movement speed of 10 mm/min. Then, for the same condition, the load was applied cyclically (10 cycles) with a higher speed on the order of millimeters per second, as shown in Table 3.

**Figure 3:**
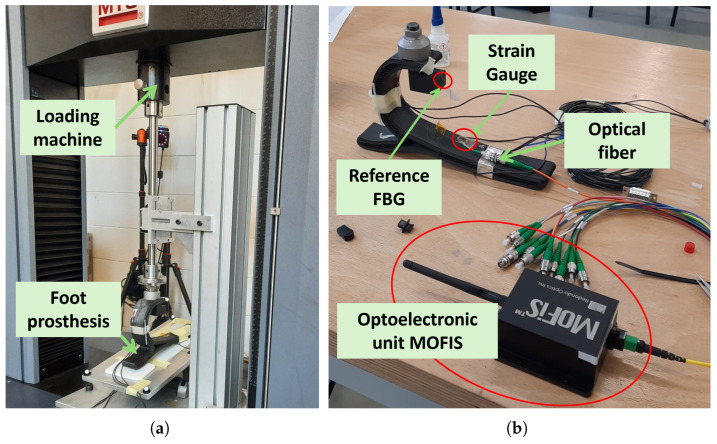
Set-up implemented to perform sensor validation tests: (**a**) Focus on the MTS loading machine and the foot prosthesis. (**b**) Focus on the FBG-based monitoring system highlighting the MOFIS*^TM^* optoelectronic unit, the reference FBG sensor, and the upper portion of the strain gauge, the sensing FBG is on the prosthesis sole in correspondence with the strain gauge as shown in Figure 2a.

Figure 3 shows the setup involved during the tests, which included:

- the foot prosthesis;
- the FBG-based monitoring system composed of the fiber with the sensing and the reference FBGs, the optoelectronic unit MOFIS*^TM^*, a processing unit;
- the strain gauges, the signal amplifier SCOUT55, a processing unit;
- the MTS compression machine provided by a platform to allow prosthesis inclination during the tests.

## 3. Experimental Results and Discussion

### 3.1. Compensation Variable Identification and Hypothesis Verification

The Pearson coefficient computed in (6b) is $\rho_{Hp1}=0.99$, confirming the hypothesis **Hp1** of linearity between the drift signals in the sensing and reference detection channels.

The result of the least-square-based identification method applied to (11) for the definition of the compensation variable k is shown in (13):

(13) k=0.14

The identified compensation variable applies to other scenarios or materials if the selected FBGs are compatible with detection channel four for the sensing FBG and detection channel one for the reference FBG, as the ones selected in the presented study.

**Figure 4:**
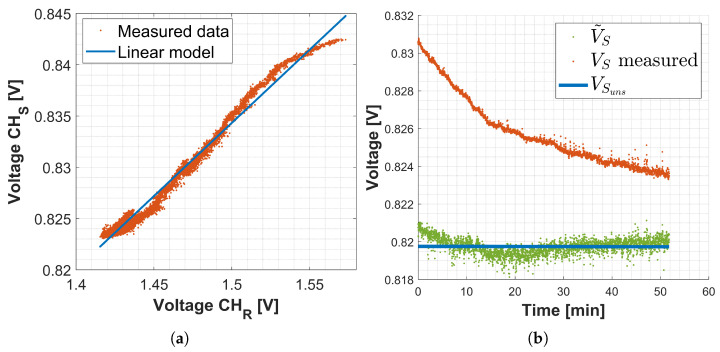
(**a**) Identification of parameter ***k***: the orange dots represent the dataset used to identify the compensation variable ***k***, whereas the blue line shows the drift on the sensing channel estimated using the identified linear model. (**b**) Effect of the drift compensation.

The parameter ***b*** is calculated during the identification process, but it is not necessary to solve (9). However, ***b*** can be considered a calibration parameter. According to **Hp2**, its value depends on the measured voltages at t=0, as demonstrated in (14). Equations (14a) and (14b) show the correctness of **Hp2** (7) and (8):

(14a) $$b_{identified}=0.62\;V$$

(14b) $$\begin{array}{cc} b_{exp} & ={\tilde{V}}_{S}\left(\epsilon ,\Delta T\right)+V_{S_{offset}}-\\ \; & \;-k\cdot \left({\tilde{V}}_{R}\left(\epsilon ,\Delta T\right)+V_{R_{offset}}\right)=\\ \; & =V_{S_{uns}}-k\cdot V_{R_{uns}}=0.63\;V \end{array}$$

where $b_{identified}$ is the parameter ***b*** identified on the dataset of Figure 4a, and $b_{exp}$ is the one obtained imposing **Hp2** and using experimental data. In Figure 4a, the y-axis represents the data from the sensing channel, while the x-axis represents the data from the reference channel. The orange signal corresponds to the dataset used during the compensation variable identification procedure. In contrast, the blue straight line represents the model derived from the identification process. In particular, the blue line was obtained by inputting the drift component of the signal acquired from the reference FBG into the model, which then estimated the drift in the signal in the sensing channel.

Applying (9), reported below for clarity, and considering the identified parameter ***k*** (13), a constant signal is expected: in this case ${\tilde{V}}_{S}\left(\epsilon ,\Delta T\right)$ is equal to $V_{S_{uns}}$ because the experiment is performed with no strain or temperature variation on the FBG.

(15) $$\begin{array}{cc} {\tilde{V}}_{S}\left(\epsilon ,\Delta T\right) & =V_{S_{uns}}=\\ \; & =V_{S_{meas}}-V_{S_{offset}}-k*\left(V_{R_{meas}}-V_{R_{uns}}\right) \end{array}$$

Figure 4b shows the process just explained: the orange signal is the measured voltage that is affected by the drift. The green signal is the ${\tilde{V}}_{S}$ signal, so the acquired voltage after drift compensation. As expected ${\tilde{V}}_{S}$ is constant and equal to $V_{S_{uns}}$, the blue strength line.

### 3.2. Algorithm Validation

**Figure 5:**
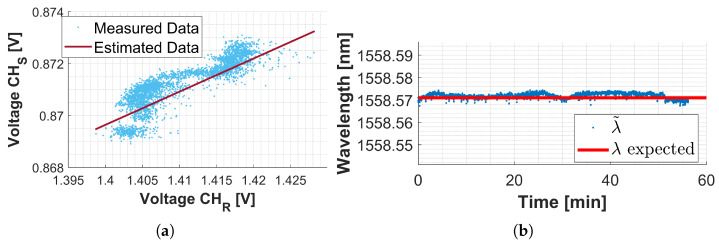
Validation of the identified linear model. (**a**) Comparison between the measured data (light-blue dots) and the estimated data (bordeaux straight line). (**b**) Resembles between the wavelength expected value and the $\lambda_{expected}$ (red line) and the wavelengths $\tilde{\lambda }$ obtained from the experimental data by applying the voltage to the wavelength conversion model after the drift compensation procedure was completed.

To evaluate the goodness of the identified linear model, the algorithm was tested on a dataset different from the one used to perform identification. The validation dataset was obtained following the same guidelines used for identification datasets and described at the beginning of Section 2.5.1. In Figure 5a the light-blue signal is the dataset used in validation, while the bordeaux straight line represents the estimated data obtained using the identified linear model.

Estimated data:

(16a) $$V_{S_{estimated}}=k*V_{R_{meas}}+b_{exp}$$

(16b) k=0.14

(16c) $$b_{exp}=V_{S_{uns}}-k*V_{R_{uns}}$$

Once the drift is compensated, the informative voltage share can be transformed into wavelength using the conversion model. Figure 5b shows in blue the wavelength obtained from experimental data after drift compensation. In red is represented the wavelength of the unstretched FBG, which is the expected one.

The applied procedure validated the effectiveness of the developed compensation algorithm. Before applying the compensation procedure, wavelength drifts caused an error in the measured wavelength of 243 pm. In [19], it was demonstrated that the overheating of the interrogation devices is the main cause of wavelength drift. Over the years, different solutions have been proposed. Some studies were focused on hardware solutions, such as applying a thin-film dielectric reflection filter on the fiber end. This method utilizes the temperature dependency of the material’s spectral characteristics. Specifically, comparing the wavelength reflected by the grating with that reflected by the interference filter allows for compensation [36]. The solution initially developed for temperature referencing also addresses wavelength drift caused by light source instability. In [37], interferometric drift is managed with passive compensation to ensure stable measurements. The reference system is based on two FBG sensors, one mounted on a glass substrate and the other on an aluminum substrate. In [22], an effective solution is proposed, similar to the solution in the present work, based on two FBGs, one used as a sensor and the other as a reference. The developed model incorporates drift as an additional component to the standard Formula (2), and experimental results validate its effectiveness. This solution proves to be simpler compared to the one proposed for the MOFIS*^TM^* interrogation device. However, due to the nonlinear relationship between output voltage and intensity in our case, it is not feasible to model and compensate for the drift as they proposed. An interesting approach to this problem is the use of machine learning techniques to overcome wavelength drift issues. In [21], a Convolutional Neural Network was implemented to predict the wavelength drift error, and a Long Short-Term Memory neural network was used to correct the wavelength drift error in the demodulation of the Fiber Fabry-Pérot Tunable Filter, achieving a 96% improvement in error. The same problem is addressed in [38] by integrating an active feedback system based on a PID controller that acts on the heating/cooling of the laser to maintain stability at the wavelength.

#### Temperature Test

**Figure 6:**
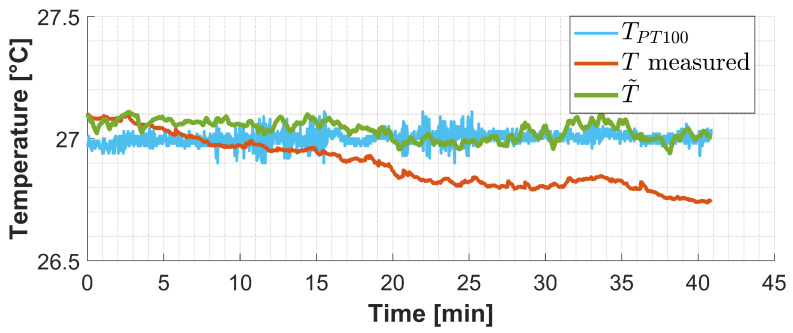
Static temperature test. In light blue ($T_{PT100}$) is the temperature measured by the reference PT100 sensor. In orange ($T_{measured}$) is the temperature measured by the FBG sensing system obtained without performing the drift compensation procedure. In green ($\tilde{T}$) is shown the temperature measured by the FBG sensing system obtained by applying the proposed compensation algorithm.

Figure 6 shows the results obtained from the temperature test. The light-blue signal is the temperature measured by the PT100 sensor inside the thermal box. The orange signal is the temperature measured by the sensing FBG and obtained without performing drift compensation. Instead, the green one is the sensing FBG temperature calculated after completing drift compensation on the measured voltage. The effect of the drift and the related measurement error becomes particularly evident after 10 min of acquisition.

To quantify the goodness of the drift compensation, the error between the PT100 measured temperature and the compensated FBG temperature ($\tilde{T}$) and the error between the PT100 measured temperature and the uncompensated FBG temperature (*T* measured) are evaluated and compared. The following indexes are computed:

- Root Mean Square Error (RMSE);
- Mean Absolute Error (MAE);

Table 4 resumes these parameters and the obtained improvement.

The results show that the developed compensation algorithm effectively overcame the wavelength drift problem allowing it to achieve long-term stability. Wider temperature ranges were not investigated because the drift phenomenon is intrinsically associated with the optoelectronic unit rather than the FBG itself. The relationship between drift and signal remains consistent regardless of the temperature to which the sensing element is exposed.

In [39], the stability goal was reached by integrating a magneto-optic wavemeter into the interrogation system, resulting in a temperature accuracy of 0.1 °C. A stability test was also performed in [37], demonstrating the effectiveness of their proposed approach. The compensation algorithm presented in this work appears to be more feasible as it does not require additional hardware or complex tuning of a feedback controller. However, it has the drawback of requiring a reference FBG.

Table 5 provides a comparison between the performance of the proposed drift compensation algorithm (second column) and the performances of other studies addressing similar issues (third column). In the first column, the performance metrics under evaluation are reported. The results demonstrate that the performance of the proposed method is consistent with that of existing approaches.

### 3.3. Mechanical Test

**Figure 7:**
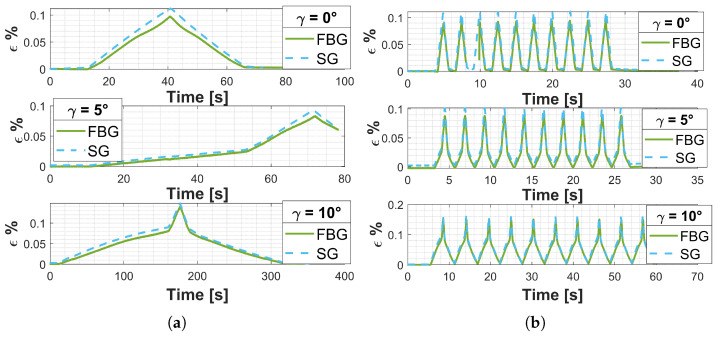
Induced deformation for positive tilt angles. (**a**) Quasi-static tests. (**b**) Dynamic tests.

The percentage strain (ϵ%) was obtained using the conversion formula (3). The results are reported in Figure 7a for the quasi-static tests and Figure 7b for the dynamic tests, specifically, the continuing line refers to the FBG sensor measure, and the dashed line refers to the strain gauges measure. For positive tilt angles configuration, $\gamma =5^{\circ }$ and $\gamma =10^{\circ }$, the foot prosthesis is supported on the toe, and the heel is detached from the platform. During the test, the compression leads the heel to impact the platform. The time instant the heel impacts the platform corresponds to the change in the curve slope, which appears clearly both in quasi-static and dynamic tests.

The Root Mean Square Error (RMSE) and the Absolute Mean Error (MAE) between the percentage strain measured by the strain gauge and the one measured by the FBG are the metrics selected to evaluate the performance of the implemented monitoring system based on FBG sensors. Table 6 shows the results.

By expressing the average values of RMSE and MAE as a percentage of the maximum recorded strain value, the results of (17) and (18) are obtained.

(17) $$PercentageErrorMAE=\frac{MAE_{MeanValue}}{\epsilon {\%}_{max}}*100=3.27\%$$

(18) $$PercentageErrorRMSE=\frac{RMSE_{MeanValue}}{\epsilon {\%}_{max}}*100=5.09\%$$

**Figure 8:**
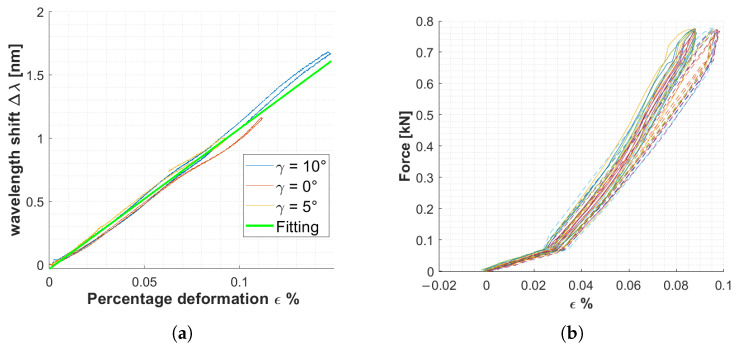
(**a**) Fitting relationship between the deformation measure with SG and the related wavelength shift. (**b**) Relationship between force and strain: the solid line represents the signals acquired with FBG sensors, and the dashed line the signals measured by the strain gauge.

Figure 8a shows the relationship between the wavelength shift returned by the FBG and the percentage strain measured by the Strain Gauges. The fitting on the experimental data (19) turns out to be linear, consistent with the statements in Section 2.1:

(19) $$\Delta \lambda =m_{exp}\cdot \epsilon \%=11.1\cdot \epsilon \%$$

So the fractional wavelength change experimentally evaluated turns out to be 11.1 nm for 1% strain, which is slightly lower than the theoretical one that is 12 pm for 1% strain [40]. The bonding layer causes a reduction in the gauge factor [41] because it does not allow a strain transmission rate of 100% [42]. The gauge factor obtained is consistent with those found in similar studies; for instance, in [43], the strain sensitivity factor was determined by applying uniaxial stress to a Carbon-Fiber-Reinforced Plastics sample with an FBG sensor mounted on the surface.

Figure 8b illustrates the relationship between loads applied during cyclic tests and strain measured by the FBG sensor (solid line) and strain gauge (dashed line), respectively.

It can be observed that for the same applied load, the strain gauge measures a higher strain, with a percentage error of 9%. Considering that the strain gauge could not be placed in the same position as the FBG sensor, and given the differing dimensions of the FBG sensor and strain gauge, these differences are deemed acceptable and not indicative of any malfunction in the FBG-based measurement system. Similar findings were reported in [44], where two FBGs embedded in a glass fiber-reinforced plastic tube were used to detect strain changes under loading. The FBG results were compared to the deformation measured by resistance strain gauges surface-mounted in the middle of the tube.

## 4. Conclusions

This study implements a drift-compensated monitoring system based on FBG sensors in a 3D-printed foot prosthesis representing a significant advancement over previous work using a miniaturized and wearable optoelectronic unit (i.e., MOFIS*^TM^*). Characterized by its low weight and small size, MOFIS*^TM^* is suitable for wearable applications. However, miniaturizing the system affects the thermal stabilization of the light source, resulting in acquired signals with a decreasing trend. To ensure measurement accuracy and stability, we propose a method to compensate for the error related to wavelength drift. The proposed compensation algorithm can be applied to the acquired signals in real-time to ensure a prompt response from the monitoring system, a key feature for a wearable device.

This study represents the initial step in developing a performance monitoring system integrated into the prosthesis. This system not only enhances the safety of the prosthesis but also detects structural changes that could potentially harm the patient in the long term. This wearable and drift-compensated FBG-based monitoring system could also provide interesting information to optimize the prosthesis response during its usage. The next steps will include identifying the position of the FBG sensors and optimizing the number of sensors to acquire as much information as possible on the mechanical response of the prosthesis. In addition, it will be necessary to identify the variable through which to monitor the performance of the prosthesis. The presented approach focuses only on the compensation of drift caused by light source instability. A further development required for implementing the solution in real-world scenarios is the integration of compensation for external temperature variations. Lastly from the algorithm point of view the limitation represented by the influence of external factors, such as mechanical strain and vibrations, could be addressed by implementing a preprocessing stage based on a low pass filter on the signal acquired by the reference FBG.

## Figures and Tables

**Table 1:** MOFIS*^TM^* engineering specifications [31].

Monitoring Mode	WDM Multiplexing
Sensing Channels	Four FBG transducers on a single fiber cable network
Wavelength Range	60 nm @ 1550 nm C-Band
Sensor Dynamic Range	2000 μϵ per each FBG sensor
Sensor Accuracy	≤0.1 μϵ
Sampling Rate	≤250 Hz to 20 kHz
Data Communication	Wireless (UDP protocol)
Power Consumption	≤1250 mW @ 3.7 Vdc (Max.)
Power Supply	Li-Battery 3.7 V @ 6 W/h—Rechargeable
Weight	<250 g

**Table 2:** FBGs parameters.

	FBG_1_	FBG_2_
Wavelength ($\lambda_{B0}$)	1529 nm	1558.694 nm
Bandwidth	0.27 nm	0.292 nm
SLSR	22.25 dB	21 dB
Reflectivity	51.35%	49.88%
FBG length	5 mm	5 mm
Fiber type	Polymide A2	Polymide A2
Recoating	Polymide	Polymide

**Table 3:** Loads applied for different levels of inclination.

Tilt Angle γ [deg]	Load [N]	Speed [mm/s]
0	1100	5
5	660	20
10	660	36

**Table 4:** Indexes computed to evaluate compensation goodness.

	RMSE [°C]	MAE [°C]
$\tilde{T}$	0.06	0.05
*T* uncompensated	0.15	0.13
**Improvement**	50%	60%

**Table 5:** Comparison of performance metrics between the proposed MOFIS*^TM^* drift compensation algorithm and existing studies from the literature.

Metric	MOFIS*^TM^* Compensation Algorithm	Literature
Error % on full scale	0.18%	0.5% [37]
MAE	1.9 pm	1.81 pm [21]
Absolute error	0.03 °C	0.05 °C [22]

**Table 6:** Evaluation Metrics Table.

	RMSE [ϵ%]	MAE [ϵ%]
γ=0°	0.0093	0.0058
γ=5°	0.0071	0.0066
γ=10°	0.0080	0.0055
Mean values	0.0081	0.0059

## Data Availability

Dataset available on request from the authors.

## References

[1] McDonald C.L., Westcott-McCoy S., Weaver M.R., Haagsma J., Kartin D. (2021). Global prevalence of traumatic non-fatal limb amputation. Prosthet. Orthot. Int..

[2] Yuan B., Hu D., Gu S., Xiao S., Song F. (2023). The global burden of traumatic amputation in 204 countries and territories. Front. Public Health.

[3] Chadwell A., Diment L., Micó-Amigo M., Morgado Ramírez D.Z., Dickinson A., Granat M., Kenney L., Kheng S., Sobuh M., Ssekitoleko R. (2020). Technology for monitoring everyday prosthesis use: A systematic review. J. Neuroeng. Rehabil..

[4] Hafner B.J., Sanders J.E. (2014). Considerations for development of sensing and monitoring tools to facilitate treatment and care of persons with lower limb loss. J. Rehabil. Res. Dev..

[5] Moreno-Gomez A., Perez-Ramirez C.A., Dominguez-Gonzalez A., Valtierra-Rodriguez M., Chavez-Alegria O., Amezquita-Sanchez J.P. (2018). Sensors used in structural health monitoring. Arch. Comput. Methods Eng..

[6] Kinet D., Mégret P., Goossen K.W., Qiu L., Heider D., Caucheteur C. (2014). Fiber Bragg Grating Sensors toward Structural Health Monitoring in Composite Materials: Challenges and Solutions. Sensors.

[7] Glisic B., Inaudi D. (2007). Fibre Optic Methods for Structural Health Monitoring.

[8] Paloschi D., Polimadei A., Korganbayev S., Orsetti V., Mazzotta C., Cigada A., Caponero M.A., Saccomandi P. (2023). Three-Dimensional-Printed Sensing Samples Embedding Fiber Bragg Gratings: Metrological Evaluation of Different Sample Materials and Fiber Coatings. IEEE Trans. Instrum. Meas..

[9] Rohan R., Venkadeshwaran K., Ranjan P. (2024). Recent advancements of fiber Bragg grating sensors in biomedical application: A review. J. Opt..

[10] Al-Fakih E.A., Abu Osman N.A., Mahamd Adikan F.R., Eshraghi A., Jahanshahi P. (2016). Development and Validation of Fiber Bragg Grating Sensing Pad for Interface Pressure Measurements Within Prosthetic Sockets. IEEE Sens. J..

[11] Al-Fakih E.A., Osman N.A.A., Eshraghi A., Adikan F.R.M. (2013). The Capability of Fiber Bragg Grating Sensors to Measure Amputees’ Trans-Tibial Stump/Socket Interface Pressures. Sensors.

[12] Galvão J.R., Zamarreño C.R., Martelli C., Cardozo Da Silva J.C., Arregui F.J., Matías I.R. (2017). Mapping in Carbon-Fiber Prosthesis Using Optical Fiber Sensors. IEEE Sens. J..

[13] Butt A.M., Qureshi K.K. (2019). Smart Lower Limb Prostheses with a Fiber Optic Sensing Sole: A Multicomponent Design Approach. Sens. Mater..

[14] Lavarda M.D., Gomes D.F., Paes T., de Sousa R.O., Dreyer U.J., da Silva J.C.C., Martelli C. (2023). Smart Foot Based on FBG Integrated in Composite Material and Adaptive Fuzzy Controller. IEEE Sens. Lett..

[15] Pant S., Umesh S., Asokan S. (2018). Knee Angle Measurement Device Using Fiber Bragg Grating Sensor. IEEE Sens. J..

[16] R. Diaz C.A., Leal-Junior A.G., M. Avellar L., C. Antunes P.F., Pontes M.J., Marques C.A., Frizera A., N. Ribeiro M.R. (2019). Perrogator: A Portable Energy-Efficient Interrogator for Dynamic Monitoring of Wavelength-Based Sensors in Wearable Applications. Sensors.

[17] Ogawa K., Koyama S., Haseda Y., Fujita K., Ishizawa H., Fujimoto K. (2019). Wireless, Portable Fiber Bragg Grating Interrogation System Employing Optical Edge Filter. Sensors.

[18] Mendoza E.A., Esterkin Y., Kempen C., Sun Z. (2011). Multi-channel monolithic integrated optic fiber Bragg grating sensor interrogator. Photonic Sens..

[19] Friedemann M., Voigt S., Werner M.L., Hecker R., Mehner J. Drift analysis and stabilization of a Fiber Bragg Grating interrogation device. Proceedings of the 2020 International Conference on Applied Electronics (AE).

[20] Sheng W., Dang H., Peng G.D. (2021). Hysteresis and temperature drift compensation for FBG demodulation by utilizing adaptive weight least square support vector regression. Opt. Express.

[21] Lin H., Sheng W. Research on Temperature Drift Error Correction of Tunable Filter Based on CNN-LSTM. Proceedings of the 2023 4th International Conference on Advanced Electrical and Energy Systems (AEES).

[22] Yang Z., Yao G., Li Y. A novel wavelength calibration method for Fiber Bragg Grating sensor system. Proceedings of the 2011 International Conference on Electric Information and Control Engineering.

[23] Petermann I. (2007). Fibre Bragg Gratings: Characterization, Realization and Simulation. Ph.D. Thesis.

[24] Fernández-Ruiz M.R., Carballar A. (2021). Fiber Bragg Grating-Based Optical Signal Processing: Review and Survey. Appl. Sci..

[25] Chen J., Liu B., Zhang H. (2011). Review of fiber Bragg grating sensor technology. Front. Optoelectron..

[26] Hill K., Meltz G. (1997). Fiber Bragg grating technology fundamentals and overview. J. Light. Technol..

[27] Korganbayev S., Orrico A., Bianchi L., Paloschi D., Wolf A., Dostovalov A., Saccomandi P. (2021). PID Controlling Approach Based on FBG Array Measurements for Laser Ablation of Pancreatic Tissues. IEEE Trans. Instrum. Meas..

[28] Rao Y.J. (1997). In-fibre Bragg grating sensors. Meas. Sci. Technol..

[29] Ozcelik D., Parks J.W., Wall T.A., Stott M.A., Parks H.C.J.W., Hawkins A.R., Schmidt H. (2015). Optofluidic wavelength division multiplexing for single-virus detection. Proc. Natl. Acad. Sci. USA.

[30] Chae C.J., Oh N.H. (1998). WDM/TDM PON system employing a wavelength-selective filter and a continuous-wave shared light source. IEEE Photonics Technol. Lett..

[31] (2019). Manual-MOFIS-M400-100X.

[32] Castro-Caicedo A., Nieto-Callejas M., Torres P., Lain R., Suarez-Burgoa L. (2019). Manufacturing and reliability of a fiber Bragg grating strain sensor designed for uniaxial compression test of rocks. Dyna.

[33] Martulli L.M., Luppino G., Rollo G., Kostovic M., Romanò J., Garavaglia L., Sorrentino A., Pittaccio S., Saccomandi P., Tarabini M. Finite element simulation of the full stance-phase in the design process of a 3d-printed composite foot prosthesis. Proceedings of the 11th International Conference on Composites Testing and Model Identification.

[34] (2016). BSI Standards Publication Prosthetics—Quantification of Physical Parameters of Ankle-Foot Devices and Foot Units.

[35] (2016). BSI Standards Publication Prosthetics—Testing of Ankle-Foot Devices and Foot Units—Requirements and Test Methods.

[36] Arya V., Sherrer D., Wang A., Claus R., Jones M. (1997). Application of thin-film optical filters to the temperature compensation of optical fiber grating-based devices. IEEE Trans. Instrum. Meas..

[37] Todd M.D., Johnson G.A., Althouse B.L. (2001). A novel Bragg grating sensor interrogation system utilizing a scanning filter, a Mach-Zehnder interferometer and a 3 × 3 coupler. Meas. Sci. Technol..

[38] Chehura E., Ye C.C., Tatam R.P. (2003). In-line laser Doppler velocimeter using fibre-optic Bragg grating interferometric filters. Meas. Sci. Technol..

[39] Li Y., Lu B., Ren L., Chen H., Qiu Y., Mao B., Zhou P., Zhao C., Dong X. (2019). A highly precise FBG sensor interrogation system with wavemeter calibration. Opt. Fiber Technol..

[40] Alvarez-Botero G., Baron F.E., Cano C.C., Sosa O., Varon M. (2017). Optical sensing using fiber bragg gratings: Fundamentals and applications. IEEE Instrum. Meas. Mag..

[41] Lin Y.B., Chang K.C., Chern J.C., Wang L. (2005). Packaging methods of fiber-Bragg grating sensors in civil structure applications. IEEE Sens. J..

[42] Cheng C.C., Lo Y.L., Li W.Y., Kuo C.T., Cheng H.C. (2007). Estimations of fiber Bragg grating parameters and strain gauge factor using optical spectrum and strain distribution information. Appl. Opt..

[43] González-Gallego M., Terroba Ramírez F., Martínez-Vicente J.L., González del Val M., López-Cela J.J., Frövel M. (2024). Fiber Bragg Gratings Sensor Strain–Optic Behavior with Different Polymeric Coatings Subjected to Transverse Strain. Polymers.

[44] Chen Y., Hsieh C., Lin C. (2011). Strain measurement for composite tubes using embedded, fiber Bragg grating sensor. Sens. Actuators Phys..

